# Myopic Correction with Iris-Fixated Phakic Intraocular Lenses: Twelve-Year Results

**DOI:** 10.1155/2021/7027793

**Published:** 2021-10-07

**Authors:** Iveta Nemcova, Jiri Pasta, Katerina Hladikova, Martin Komarc, Darina Pospisilova, Pavel Nemec, Jan Tesar, Vladimir Kratky, Martin Sin

**Affiliations:** ^1^Department of Ophthalmology, Military University Hospital Prague, Prague, Czech Republic; ^2^1st Faculty of Medicine of Charles University, Prague, Czech Republic; ^3^Health Sciences Centre, Queen's University, Kingston, Canada

## Abstract

**Purpose:**

To evaluate a 12-year follow-up of myopic patients after iris-fixated phakic intraocular lenses (IF pIOLs) implantation. *Setting*. Ophthalmology Department, Military University Hospital in Prague (Czech Republic).

**Design:**

Single-center retrospective cohort study.

**Methods:**

We describe the results of a cohort study that included 85 eyes of 46 myopic patients who underwent implantation of Verisyse myopia, Veriflex, and Verisyse myopia toric (all Abbott Medical Optics, Inc.) intraocular lenses. Refractive functions and adverse events were assessed preoperatively, at 6 months, and 1, 2, 5, and 12 years after IF pIOL implantation.

**Results:**

Mean spherical equivalent was measured as −9.37 ± 2.87 D, 0.14 ± 0.61 D, and −0.42 ± 1.08 D, preoperatively, at 6 months and 12 years postoperatively, respectively. There was a significant reduction in the cylinder after surgery. At 12 years postoperatively, 90% of eyes had uncorrected distance visual acuity (UDVA) of 20/40 and 64% of 20/20. The safety index was 1.10 for the whole postoperative follow-up period. We found cataract formation in 3 eyes (3.5%). The endothelial cells loss (EC loss) directly caused by IF pIOL implantation was 6.0%, 8.10%, 12.8%, and 11.9%, at 1, 2, 5, and 12 years, respectively. In our cohort, 95% of eyes lost a higher percentage of EC than would be expected from a physiological loss at 12 years postoperatively. We found a significant negative interaction between preoperative pachymetry and EC loss, indicating that the lower pachymetry leads to a faster decline in endothelial cells density (ECD). IF pIOL re-enclavation was found in 28% of eyes. 7% of subluxations were caused by trauma. The mean time of nontraumatic re-enclavation was 6 years postoperatively.

**Conclusions:**

The study confirmed the advantages of IF pIOL implantation due to rapid visual recovery and stable visual function over the 12-year follow-up and also showed the influence of lower corneal pachymetry regarding EC loss.

## 1. Introduction

Phakic intraocular lens (pIOL) implantation has been a powerful solution for moderate and high myopia for more than 30 years. It helps patients who may have some contraindication to excimer laser surgery to be glasses independent. The first models of the pIOLs implanted in the 1980s and 1990s were angle-supported anterior chamber pIOLs. Because of unacceptable complication rate, especially corneal endothelial cells loss (EC loss) from the pIOL's proximity to the corneal endothelium, they were consequently removed from the market.

EC loss was also the problem with the Worst–Fechner iris-fixated IOLs (IF pIOLs), a coplanar single-piece PMMA pIOLs that were enclavated in the folds of midperipheral iris stroma, a relatively immobile portion of the iris. The first implantation of IF pIOL in myopic patient occurred in 1986. By the end of the 1990s, a newly designed, safer model of PMMA IF anterior chamber pIOLs was introduced. First, a foldable single-piece Artisan (Ophtec BV)/Verisyse (Abbott Medial Optics, Inc.) made from Perspex CQ-UV appeared on the market. Subsequently, a foldable variant of an IF anterior chamber pIOL Artiflex (Ophtec BV)/Veriflex (Abbott Medial Optics, Inc.) composed of hydrophobic polysiloxane was designed. The optic vault measured approximately 0.87 mm anterior from the iris and provided good clearance for the anterior lens capsule and the endothelium of the cornea [1–3].

During the past 10 years, several long-term reports about IF pIOLs were published. The EC loss and cataract formation were found to be more frequent than under physiological conditions. Detection of basic risk factors showed to be important for correct indication and minimization of postoperative complications. Most of the publications reported 5-year results [4–8], but only a few of them spanned more than 10 years [9–15]. In this paper, we show our results of a 12-year follow-up of myopic patients cohort after implantation of Verisyse myopia, Verisyse myopia toric, and Veriflex pIOL (Abbott Medical Optics, Inc.) lenses.

## 2. Patients and Methods

### 2.1. Study Design

We describe the results of our cohort study that includes a group of 85 eyes in 46 myopic patients, 13 men (28%) and 33 women (72%). The patients underwent implantation of IF pIOLs from January 2005 to November 2010, and we were tracking their refractive results and complications over 12 years. All surgeries were performed by one surgeon in the Department of Ophthalmology, Military University Hospital, Prague (Czech Republic). The study was approved by the Medical Ethics Committee at University Medical Centre in accordance with tenets of the Declaration of Helsinki.

### 2.2. Inclusion and Exclusion Criteria

We strictly adhered to the following inclusion criteria. All patients had to be older than 18 years of age and their refractive error had to be stable at least for 1 year. The IF pIOL implantation was carried out only in patients where excimer laser surgery was not indicated for the correction of existing ametropia. We respected the following criteria in the operated eye: ECD (endothelial cells density) >2300 cells/mm^2^, ACD (anterior chamber depth) from endothelium (the distance between endothelium and anterior surface of the clear lens) >2.9 mm, iridocorneal angle ≥30°, no anomaly of iris or pupil and mesopic pupil size <6 mm. One eye (1.2%) of 1 patient underwent a keratoplasty prior to pIOL implantation. Seven eyes (8.2%) in 5 patients had a previous scleroplasty procedure, and their refraction had been stable for more than 2 years before the IF pIOL was implanted. Preventative laser photocoagulation was carried out in one patient (2 eyes) to treat lattice degeneration to decrease the risk of retinal detachment.

As for exclusion criteria, we excluded glaucoma and IOP >21 mmHg, active disease in the anterior segment, recurrent or chronic uveitis, any form of cataract, preexisting macular pathology or abnormal retinal condition, as well as any systemic disease (autoimmune disorders, connective tissue disease, atopy, and diabetes mellitus) [1]. None of the patients had keratoconus.

### 2.3. Types of PIOLs, Power Calculation, and Surgical Technique

Three types of IF pIOLs were implanted. In myopic patients with the cylinder under 2 dioptres, we used the PMMA Verisyse myopia (Abbott Medical Optics, Inc.) in the period 2005–2008 and the foldable Veriflex pIOL (Abbott Medical Optics, Inc.) in the period 2008–2010, 40 eyes (57%) and 28 eyes (33%), respectively. In 17 eyes (20%), myopic eyes with cylinder higher than 2 dioptres PMMA Verisyse myopia toric (Abbott Medical Optics, Inc.) was implanted.

The pIOL power calculation was carried out before lens implantation. The commonly used IF pIOLs calculator worked with Van der Heijde nomogram [1]. It was based on keratometry, ACD, and the best spectacle correction and was axial length independent.

We proceeded with standard surgical technique as described by Güell et al. [1] based on the type of IF pIOL. In all cases, we used a scleral incision secured by an infinity suture in the end.

### 2.4. Outcome Measurements

Our data were collected preoperatively, at 6 months, and at 1, 2, 5, and 12 years after the IF pIOL implantation. All preoperative data are given in Table 1.

Firstly, we concentrated on refractive outcomes after IF pIOLs implantation. The objective refraction was measured out on the autorefractor (Nidek) and subjective refraction and visual acuity on the Snellen projection chart (Nidek). We compared preoperative corrected distance visual acuity (CDVA) with postoperative CDVA and uncorrected visual acuity (UCVA). We evaluated spherical equivalent (D) and cylinder (D) postoperatively and formed Efficacy Index (EI) and Safety Index (SI).

Secondly, we concentrated on intraocular pressure changes (measured by noncontact tonometer, Topcon) and adverse events after the IF pIOLs implantation, such as cataract formation, traumatic or spontaneous luxation, and subluxation. The latter was solved by reposition or re-enclavation of IF pIOL. We checked for a presence of retinal detachment. Patients were also asked regarding subjective problems with glare and halo phenomena.

Thirdly, we focused on ECD and EC loss which was carried out by one type of endothelial microscope (CSO) during the whole period. We carefully observed baseline topometric parameters such as ACD, keratometry, pachymetry (Pentacam, Oculus), the axial length of the globe (IOL Master 500, Zeiss), and compared them with postoperative follow-up ECD to identify a possible cause of long-time EC loss.

### 2.5. Statistical Analysis

In the first step, we computed basic descriptive statistics of central tendency (mean, median, percentage), dispersion (variance, standard deviation), and shape (kurtosis, skewness) for all variables under study. A linear mixed-effects model was applied to study the longitudinal changes in refractive results, intraocular pressure, and EC loss. In these models, the repeated measures during the study period were nested within each eye, and an unstructured covariance matrix was used to model the relationships amongst the observed variables. We next extended the model by including time-invariant covariates (gender, degree of myopia, keratometry, ACD, pachymetry) to examine the effects of baseline “risk factors” on longitudinal changes in ECD. The level of statistical significance was set at *α* = 0.05. Statistical analyses were performed using IBM SPSS version 25.0 (Chicago, IL).

## 3. Results

### 3.1. Refractive Results

The mean refractive spherical equivalent (MRSE) of cohort patients was −9.37 ± 2.87 [−16,75; −2] D and mean cylinder 1.37 ± 1.44 [0; 6.75] D. Postoperative MRSE and cylinder are shown in Table 2. Six months after the surgery, the refractive result was excellent, with MRSE in mild hyperopia, 0.14 ± 0.61 D. The MRSE was stable 1 year after the surgery, but between 2 years (−0.06 ± 0.67 D), 5 years (−0.34 ± 0.84 D), and 12 years (−0.42 ± 1.08 D) after the surgery, there was a statistically important shift into myopia, with *p* values of *p* < 0.05, *p* < 0.01, and *p* < 0.05, respectively.

There was a statistically significant reduction of cylinder 6 months after IF pIOL implantation, 0.70 ± 0.48 D (*p* < 0.01). The cylinder was stable 2 years after surgery. There was a statistically significant change 5 years after the surgery of 0.86 ± 0.51 D (*p* < 0.05). 12 years after the implantation, the cylinder was again without statistically significant changes compared to the previous value.

Uncorrected (UDVA) and corrected (CDVA) distance visual acuity, efficacy index (EI), and safety index (SI) are common parameters to assess the effect of the iris-claw IOLs implantation. Visual acuity was measured in the Snellen decimal scale and is summarized in Table 3. The results showed that the mean UDVA 6 months postimplantation (0.94 ± 0.15) was statistically significantly higher than mean CDVA (0.91 ± 0.17) before the surgery (*p* < 0.05). The mean postoperative CDVA (0.98 ± 0.12) was also statistically better than the mean preoperative CDVA values (*p* < 0.01). The follow-up mean CDVA results showed statistically significant improvement 5 years after the surgery, 1.00 ± 0.07 (*p* < 0.05). With respect to UDVA, we noted statistically significant worsening between 5 and 12 years after the surgery, 0.93 ± 0.17 and 0.86 ± 0.21, respectively (*p* < 0.01).

We had to proceed with excimer laser surgery (photorefractive keratectomy-PRK) for residual refractive error in 1 eye (1.2%), one year postoperatively. There was a progression of myopia >−1.0 D in 3 eyes (3.5%) of 2 patients.

**Figure 1:**
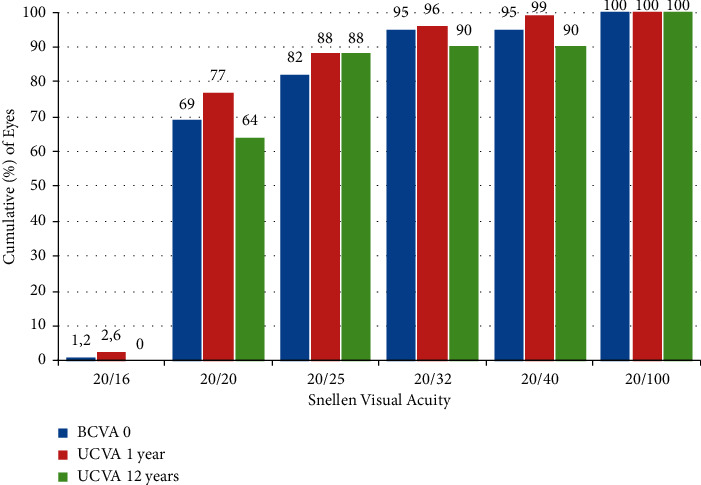
Cumulative distance visual acuity.

Efficacy is commonly reported as the cumulative percentage of eyes within the visual acuity range [14, 16]. The pooled median of the percentage of myopic eyes with a UDVA 20/40 or better at 1, 2, 5, and 12 years was 99%, 98%, 96.0%, and 90%, respectively. The pooled median of the percentage of myopic eyes with a UDVA 20/20 or better at 1, 2, 5, and 12 years was 77%, 72%, 75%, and 64%, respectively (Figure 1). The Efficacy index (EI) reflects the ratio between preoperative CDVA and postoperative UDVA (mean postoperative UDVA)/(mean preoperative CDVA) [13]. The pooled median EI was 1.06, 1.03, 1.03, and 0.96 at 1, 2, 5, and 12 years after surgery, respectively (Table 4).

**Figure 2:**
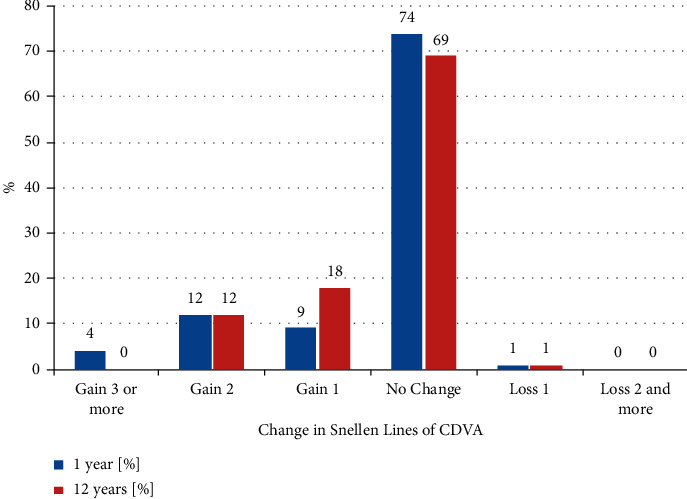
Change in corrected distance visual acuity.

Safety is commonly reported as the change in visual acuity preimplantation vs. visual acuity postimplantation [14, 16]. In our series, 99%, 98%, 99%, and 99% of eyes had stable or gain in CDVA at 1, 2, 5, and 12 years after IF pIOL implantation (Figure 2). The Safety index (SI) is defined as the ratio of (mean postoperative CDVA)/mean preoperative CDVA) [4]. The pooled median SI at 1, 2, 5, and 12 years of follow-up was 1.10, 1.10, 1.10, and 1.10, respectively (Table 4).

### 3.2. Intraocular Pressure

The incidence of secondary angle-closed glaucoma (SACG) was 4.7% in the early postoperative period. Due to the intraocular pressure elevation, we had to enlarge iridotomies in 2 eyes (2.3%) of one patient just after the primary IF pIOL implantation and in one eye (1.2%) 2 months after the IF pIOL re-enclavation. One eye (1.2%) experienced an attack of acute pupillary block glaucoma on the first night after the surgery. Iridotomies had to be enlarged 20 hours after the surgery, but pupilloplegia remained. It was rectified later with a pupilloplasty procedure. There was no pigment dispersion glaucoma in our cohort.

From the long-time perspective, the patients remained without statistically significant changes in intraocular pressure after the IF pIOLs surgery. This is supported by the values before and 5 years after the implantation, of 14.8 ± 3.0 mmHg and 15.2 ± 3.0 mmHg, respectively (*p*=0.957). All values are summarized in Table 5.

### 3.3. Adverse Events

High myopia is considered an important risk factor in peripheral retinal degeneration and the subsequent development of retinal detachment [17]. Prophylactic laser barrage treatment was used in 2 eyes (2.3%) of 1 patient. This method seems to be an effective way to prevent retinal detachment (RD) in these patients because 12 years after surgery, we did not register any cases of this complication.

One of the most frequent complications was the improper position of the phakic intraocular lens in some cases. Repositioning of the IF pIOL may be necessary due to the inadequate surgical fixation or due to the inadequate fixation after trauma [14, 18]. Overall, IF pIOL reposition or re-enclavation had to be carried out in 24 eyes (28%) of 15 patients. In 6 eyes (7%) of 6 patients the luxation of IF pIOL was caused by trauma. The mean time of nontraumatic re-enclavation was 6 years after the IF pIOL implantation.

Obviously, the most important reason for the explantation of an IF pIOL was cataract formation [4]. The incidence of cataract was 3 eyes (3.5%) of 2 patients, and it appeared at 9, 10.5, and 12 years postimplantation in our cohort.

The second most important reason for IF pIOL explantation is a high EC loss [4]. We had to carry out one pIOL explantation (1.2%) for corneal endothelium decompensation after 14 years. This patient then underwent Descemet Stripping Automated Endothelial Keratoplasty (DSAEK) and refractive lens exchange. Both surgeries went smoothly without complication, and the final UCVA was 20/20. In the end, the patient had to use glasses only occasionally for reading.

Subjective satisfaction after the IF pIOL implantation was high, and there occurred only minimum bothersome phenomena such as glare or halo. This problem was found in 2 patients with 5 mm diameter of optics in toric IF pIOLs in 2 eyes (2.3%) and with iridotomy in 1 eye (1.2%). Any sign of optics decentration did not occur, and there was no need of miotic eye drops application.

### 3.4. Endothelial Cell Loss

**Figure 3:**
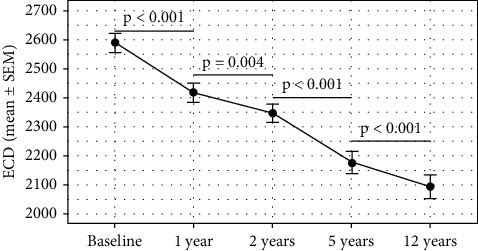
Mean endothelial cell density (ECD, cells/mm^2^) from the preoperative status to 12-year postoperative status in eyes implanted with iris-fixated phakic intraocular lenses.

The mean preoperative ECD value was 2588 ± 285 cells/mm^2^. We recorded ECD at 1, 2, 5, and 12 years after the implantation, with values of 2430 ± 312 cells/mm^2^, 2369 ± 262 cells/mm^2^, 2175 ± 298 cells/mm^2^, 2091 ± 312 cells/mm^2^, respectively (Figure 3). We computed total chronic EC loss and corrected it for a physiological EC loss of 0.6% per year (3% and 7.2% after 5 and 12 years, respectively) [14, 19]. This EC loss, directly caused by IF pIOL presence, was 6.0%, 8.1%, 12.8%, and 11.9%, at 1, 2, 5, and 12 years after IF pIOL implantation, respectively. All these EC loss values were statistically significant (Table 6).

During the postoperative 5- and 12-year follow-up, 93% and 95% of the eyes lost a higher percentage of EC than the expected physiological loss, respectively, in our cohort. According to the AAO Task Force guideline for standardized reporting on EC loss in studies of pIOLs [9, 20], we report a percentage of eyes with ≥25% EC loss. In the period 5 and 12 years after the IF pIOL implantation, it was 15% and 20% of eyes, respectively. Only 44% of eyes, which lost >25% of endothelial cells, needed a re-enclavation of pIOL after spontaneous subluxation or trauma. On the other hand, just 6.6% and 9% of eyes after the IF pIOL reposition or re-enclavation had ≥25% EC loss at 5 and 12 years after the IF pIOL implantation, respectively. In the follow-up, 3 eyes (3.5%) of 2 patients were noted to have ECD <1500 cells/mm^2^ 12 years after IF pIOL implantation, one of these eyes (1.2%) suffered trauma and underwent subsequent re-enclavation.

Next, we tested the association between baseline risk factors and long-time EC loss. We found a significant inverse relationship between preoperative pachymetry and EC loss (*p*=0.006), indicating that the lower pachymetry leads to a faster decline in ECD. The longitudinal decrease in ECD was not significantly related to gender (*p*=0.425), degree of myopia (*p*=0.449), or keratometry (*p*=0.520). Higher baseline ACD values have shown to be indicative of slower EC loss. However, the effect was slightly above the selected significance level (*p*=0.057).

## 4. Discussion

Three different types of IF pIOLs were implanted and evaluated together in our study. Yasa and Ağca referred to no significant difference of result between particular IF pIOLs (Verisyse and Veriflex group) [21].

### 4.1. Refractive Results

Refractive stability for at least 1 year is one of the main conditions before pIOL implantation [1]. In our center, we preferred stability at least 2 years to get the best results. We preferred patients older than 20 years of age, with one exception of an 18-year old patient with bilateral progressive myopia, who also underwent surgery.

Because we worked with younger patients, we calculated IF pIOL on the side of slight hypermetropia. There was a statistically significant shift into myopia 12 years after surgery, −0.42 ± 1.08 D. There was also a statistically significant reduction in the cylinder at 6 months after IF pIOL implantation. For the rest of the 12 years postoperatively, the cylinder remained stable without statistically significant changes.

We proceeded with excimer laser correction of residual refractive error in one eye (1.2%) at 1 year postimplantation. We elected PRK because Güell et al. hypothesized that laser in situ keratomileuses in an eye with anterior chamber or IF pIOL might induce contact between the corneal endothelium and the pIOL when the microkeratome (or applanation of femtosecond laser at the present time) is used [11, 22].

We closely monitored the efficacy of this method. UDVA of 20/40 or better was found in 99% of patients at 1 year and 90% of patients at 12 years after surgery. UDVA of 20/20 or better was noted in 77% of patients at 1 year and 64% of patients 12 years after surgery. The EI remained stable at 12 years after IF pIOL implantation. The safety of IF pIOL implantation was high (99%) and stable at 12 years after surgery. Just one eye (1.2%) lost one Snellen line of BDVA. The postoperative value of the SI was 1.10 and was also stable for 12 years postoperatively.

Our results are in good agreement with other studies which showed successful refractive results of IF pIOLs implantation [7, 10, 11, 14, 23, 24]. Tahzib et al. reported the MRSE −0.7 ± 1.0 D after 10 years, with no significant change in MRSE between 1, 6, and 10 years. UDVA 20/40 or better was reached in 82% of eyes, BDVA 20/40 or better in 93.3% of eyes, and only 2.6% of eyes lost more than 2 Snellen lines of BDVA [10]. Monteiro et al. calculated 6-year post-IF pIOL implantation EI and SI, 0.94 and 1.15, respectively [24]. Jonker et al. mentioned the mean myopization −0.79 D over 10 years after surgery. UDVA 20/40 or better was found in 96% of eyes, and 7% of eyes lost 2 or more lines of CDVA. They found the explanation in higher (7.6%) incidence of eyes requiring cataract surgery [11].

### 4.2. Intraocular Pressure

There is a danger of secondary glaucoma due to the pigment dispersion or pupillary block in the early postoperative period [14]. The pigment dispersion is likely caused by abnormal pressure on the iris [25, 26]. Baikoff et al. and others reported the occurrence of pigment dispersion typically in hyperopic eyes [14, 25]. On the opposite side, Monteiro et al. found pigment precipitates in 10.17% of myopic eyes in the early postoperative period and treated them successfully with topical steroids [24]. We did not register any case of pigment dispersion in our myopic group of patients.

To prevent pupillary block, an iridotomy or iridectomy is done in the eyes with IF pIOLs. Like Monteiro et al. [24], we had one case of severe acute hypertension and pupillary block (Urrets-Zavalia syndrome) [27, 28, 29], which resulted in secondary sphincter atrophy and permanent mydriasis.

In keeping with the literature, we did not find any long-term statistical change of intraocular pressure after surgery [5, 10, 14, 24, 27].

### 4.3. Adverse Events

Jiang et al. [17] found the incidence of the RD after pIOLs implantation low and without any significant difference from the natural history of RD in highly myopic eyes. We did not record any incidence of RD over 12 years postimplantation.

Subluxation of IF pIOLs can occur spontaneously or after trauma. Spontaneous haptic disenclavation is usually connected with iris depigmentation and iris atrophy [18]. Peres-Santoja et al. [30] found iris atrophy near the enclavation site of both haptics in 81.0% of cases, but he made re-enclavation only in 9.3% of cases. Budo et al. [5] and Moran et al. [31] reported repositioning of IF pIOL in 2% of cases between 4 and 11 years postoperatively. In contrast with these reports, we carried out re-enclavation in 21% of cases. The reasons were iris stroma atrophy and poor fixation of the IF pIOL. The average time of nontraumatic re-enclavation was 6 years after IF pIOL implantation. 7% of repositioning was done for IF pIOL luxation caused by trauma. There are conflicting results in the literature regarding EC loss after primary IF pIOL enclavation and subsequent re-enclavation after subluxation of IF pIOLs. Menezo et al. [32] described 30.5% EC loss at six months after traumatic IF pIOL subluxation. In the contrary, De Sanctis et al. [33] and Titiyal et al. [18] found long-term EC loss after traumatic subluxation and repositioning of IF pIOL comparable to the EC loss after uneventful pIOL implantation. We used a special technique of re-enclavation with a 2.2 mm main corneal incision and 2 side paracentesis and did not record any statistically significant subsequent EC loss after the procedure.

Cataract development has been noted after IF pIOLs implantation. Several factors may be involved including surgical trauma, age, pIOL-crystalline lens touch (including intermittent contact during accommodation), myopia, the biocompatibility of the pIOL, change in the blood-aqueous barrier, and chronic subclinical inflammation [34]. Most cataracts reported after IF pIOL implantation were of the nuclear type [34]. Alio et al. also described that almost half of the cases of IF pIOL explantation were caused by nuclear cataract formation, and the mean time to cataract formation was 9.19 years [14, 35]. Menezo et al. reported an incidence of cataracts of 3%, and the mean time of cataract extraction was 11.4 years [36]. Consistently, the incidence of cataract formation was 3.5% in our study, and cataract extraction was performed after a mean period of 10.5 years. On the other hand, Jonker et al. explanted 10% of IF pIOLs because of cataract formation after a mean of 8 years. They cited a higher preoperative age as a risk factor for cataract formation [11]. Duignan et al. [37] summarized that removal of IF pIOLs was necessitated most frequently by cataracts (followed by endothelial cells loss). He stressed that explantation with concurrent phacoemulsification is a safe procedure with good visual outcomes. This procedure will be more frequent in the future as more patients with pIOLs reach the age of cataracts.

### 4.4. Endothelial Cell Loss

As in many other studies, our inclusion criteria were based on a minimum amount of preoperative ECD [3, 5, 6, 9, 10, 16]. Recently published studies described the minimum threshold of the preoperative ECD according to age [2]. Jonker et al. used ECD >2800 cells/mm^2^ for patients aged 21 to 25 years, >2650 cells/mm^2^ for patients aged 26 to 30 years, >2400 cells/mm^2^ for patients aged 31 to 35 years, >2200 cells/mm^2^ for patients aged 36 to 45 years, and >2000 cells/mm^2^ for patients aged more than 45 years [9]. These criteria are more strict than those that were previously recommended by Gűell et al. and others [1, 3, 7, 16].

We found that the total chronic EC loss that was significantly higher than the expected physiological dropout. We measured corrected EC loss to demonstrate the EC loss directly caused by the IF pIOL presence. The values were 6.0%, 8.1%, 12.8%, and 11.9%, at 1, 2, 5, and 12 years postoperatively. During the postoperative 5- and 12- year follow-up, 93% and 95% of the eyes lost a higher percentage than the expected physiological loss, respectively. In the period 5 and 12 years after IF pIOL implantation, 15% and 20% of eyes had an EC loss of ≥25% of the preoperative value, respectively. In the 12-year follow-up, we noticed an ECD of <1500 cells/mm^2^ in 3% of cases. We had to carry out one IF pIOL explantation (1.2%) due to corneal endothelium decompensation 14 years after the initial surgery.

Jonker et al. calculated the total chronicle EC loss in 507 eyes of 289 patients receiving the Artisan myopia® or Artisan Toric ® (Ophtec B.V.) IF pIOL. Like us, he reported the EC loss corrected for physiologic EC loss (0.6% per year), at 5.2% and 7.5% from 6 months to 5 years, and 10.9% and 15.8% from 6 months to 10 years, respectively. Ten years after implantation, ECD had decreased by ≥ 25% in 7.9% and 6.3%, whereas the ECD was <1500 cells/mm^2^ in 3.9% and 4% in the myopic and toric group. In 6% of eyes in the myopic group and 4.8% of eyes in the toric group, excessive EC loss or corneal decompensation resulted in IF pIOL explantation after 11.9 and 7.4 years, respectively [9]. Tahzib et al. found out EC loss 8.86% in a group of 89 eyes of 49 patients 10 years after Artisan® pIOL implantation [10]. Worst–Fechner et al. spoke about a statistically significant decrease of ECD in 13.4% of 127 IF pIOL implanted eyes and further four eyes undergoing penetrating keratoplasty 8 years after the pIOL implantation [13]. Galvis et al. mentioned a group of 67 eyes with implantation of an Artisan® pIOL. During the 9-year postoperative follow-up of the myopic group, 60.8% of the eyes lost a higher percentage of ECD than was physiologically expected. 3% of eyes had a final cell density of fewer than 1200 cells/mm^2^ and 1 phakic lens was explanted due to a severe decrease of the endothelial density (862 cells/mm^2^) [15]. A Paired-eye comparison of corneal endothelial cell counts after unilateral IF pIOL implantation was reported by Morral et al. [12]. The patients had implantation in 1 eye and refractive surgery (Group 1) or no surgery (Group 2) in the following eye. Both groups comprised 29 patients. The mean EC loss was 6.41% (Group 1, IF pIOLs), 5.59% (Group 1, corneal refractive surgery), 7.84% (Group 2, IF pIOLs), and 6.74% (Group 2, no surgery). He concluded that IF pIOL implantation did not produce significant EC loss up to 10 years after surgery compared with corneal refractive surgery and unoperated eyes when strict inclusion criteria were met. The limitation of this study was that a lot of patients dropped out of postoperative follow-up. Moreover, there was high intraindividual variability and some patients presented with high anisometropia (one eye was hyperopic while the following eye was highly myopic) [12].

Some papers try to predict the mean time from initial surgery to IF pIOL explantation. Bouheraoua et al. performed a linear model analysis of the 5-year follow-up to present a model that describes endothelial cell loss as a linear decrease. This model predicted that for patients with preoperative ECD of 3000, 2500, and 2000 cells/mm^2^, a critical ECD of 1500 cells/mm^2^ will be reached at 39, 28, and 15 years after the implantation, respectively [7]. Such a model seems to be very useful and helps us to improve the inclusion criteria. However, the question is whether the EC loss is really linear. We measured the corrected EC loss decelerated between 5 and 12 years after the IF pIOL implantation, at 12.78% and 11.86%, respectively. Fechner, who was together with Worst involved in the design and clinical application of the IF pIOLs in the 1980s, published a 2010 case report of late severe endothelial loss in the myopic patient between 12 years and 20 years after IF pIOL implantation. This patient had preoperatively a very rich endothelial layer and baseline ACD was more than 3.8 mm measured from corneal surface to surface of the natural lens. He pointed that the cause of the decrease of ECD was not clear and increased eye rubbing connected with age-related dry eyes was one of the possibilities [23].

As we mentioned above, the general aim is to evaluate each patient for basic risk factors to avoid unexpected postoperative EC loss. We tested the correlation between postoperative EC loss and basic factors like gender, degree of myopia, keratometry, pachymetry of the cornea, and ACD. In line with some previous studies, we did not find any significant relationship between gender, age, degree of myopia, and either basic keratometry [6]. Moreover, we did not find any significant relationship between preoperative ACD (the most discussed risk factor) and follow-up EC loss. This fact was supported by some studies [10, 15, 24], but others found a significant inverse correlation between these two parameters [6, 9, 13]. Because there was the effect of ACD only slightly above the selected significance level in our cohort, we can predict that the higher baseline ACD values are indicative of slower EC loss. Some papers presented the idea that the age of patients leads to a more shallow anterior chamber, resulting in intermittent contact between the IF pIOL and the posterior corneal surface [23]. On the contrary, Jonker et al. wrote about fluctuating ACD rising from greater accommodative capacity in younger patients [9]. But in the end, no author proved the impact of age on long-time EC loss after the IF pIOLs implantation [6, 9, 10, 15, 24].

Our outcomes describe, for the first time, the inverse correlation between the baseline corneal pachymetry and the EC loss at 12 years postoperatively. We noted slower EC loss in patients with thicker corneas and faster EC loss in those with thinner corneas. The most likely explanation of this finding could be just the effect of eye rubbing. The corneas with lower pachymetry have a stronger tendency to be deformed than the corneas with higher pachymetry. Shallowing of ACD could lead to contact between IF pIOL and endothelium. This finding corresponds with Galvis's suggestion [15] that the EC loss could be linked with chronic intermittent endothelial touch during ocular rubbing or pressure exerted on the eye at night, related to a particular sleeping position [15, 38, 39]. Currently, high refractive error and low corneal pachymetry belong to the most frequent contraindication to excimer laser surgery. According to our findings, we have to be very careful about recommending IF pIOLs to patients with a lower value of baseline pachymetry.

## 5. Conclusions

In conclusion, this study confirms the advantages of IF pIOLs implantation due to rapid visual recovery and stable visual function in the 12-year follow-up. This method is safe, effective, and predictable in the long term and seems to be the better alternative to laser corneal refractive surgery for younger patients with high myopia and the ability to accommodate. Patients with IF pIOLs have to be regularly monitored because of the risk of EC loss and cataract formation, which is higher than in the general population. We proved the relationship of lower corneal pachymetry to increased EC loss. In addition to keeping in mind the safety limits of ECD (corrected to the age) and ACD, we recommend focusing on corneal thickness measurement prior to IF pIOL implantation.

## Figures and Tables

**Table 1:** Baseline characteristics of patients.

Parameters at baseline	Mean ± SD [range]
No. of eyes	85
No. of patients	46
Age	29.3 ± 5.5 [18; 43]
Gender male/female (%)	28/72
Sphere (D)	9.37 ± 2.87 [−16.75; −2]
Cylinder (D)	1.37 ± 1.44 [0; 6,75]
CDVA, Snellen decimal scale	0.91 ± 0.17 [0.4; 1.13]
IOP (mmHg)	14.8 ± 3.0 [8.5; 22.5]
ACD from endothelium (mm)	3.30 ± 0.23 [2.75; 3.77]
Mean keratometry (D)	43.8 ± 1.6 [39.4; 47.1]
Mean axial length (mm)	26.99 ± 1.27 [24.99; 31.39]
Mean corneal pachymetry (*μ*m)	521 ± 31,95 [453; 585]
ECD	2588 ± 285 [1481; 3215]
*Type of implanted lens*	*No.*
Verisyse (Abbott)	40
Verisyse Toric (Abbott)	17
Veriflex (Abbott)	28

SD: standard deviation; No.: number; D: dioptres; CDVA: corrected distance visual acuity; IOP: intraocular pressure; ACD: anterior chamber depth; ECD: endothelial cells density.

**Table 2:** Refractive results.

Time after the pIOL implantation	No. of eyes	Sphere (D)	*p* value	Cylinder (D)	*p* value
6 months	80	0.14 ± 0.61 [−1.5; 1]	<0.001	0.70 ± 0.48 [0; 2]	0.001
1 year	75	0.04 ± 0.67 [−2.0; 1.5]	0.139	0.64 ± 0.48 [0; 2]	0.623
2 years	61	−0.06 ± 0.67 [−2.25; 1.25]	0.019	0.72 ± 0.67 [0; 2]	0.304
5 years	60	−0.34 ± 0.84 [−3.5; 1.25]	0.001	0.86 ± 0.51 [0; 2]	0.019
12 years	42	−0.42 ± 1.08 [−5.5; 1.50]	0.024	0.87 ± 0.55 [0; 2.5]	0.425

No.: number; D: dioptres; *p* value: probability value.

**Table 3:** Postoperative visual acuity results (Snellen decimal scale).

Time after the pIOL implantation	No. of eyes	UDVA	*p* value	CDVA	*p* value
6 months	80	0.94 ± 0.15 [0.55; 1.25]	0.040	0.98 ± 0.12 [0.65; 1.25]	0.001
1 year	75	0.95 ± 0.15 [0.50; 1.25]	0.666	0.99 ± 0.10 [0.70; 1.25]	0.281
2 years	61	0.93 ± 0.13 [0.40; 1.00]	0.972	0.99 ± 0.07 [0.75; 1.25]	0.729
5 years	60	0.93 ± 0.17 [0.30; 1.10]	0.972	1.00 ± 0.07 [0.8; 1.25]	0.020
12 years	42	0.86 ± 0.21 [0.30; 1.05]	0.005	1.00 ± 0.06 [0.9; 1.25]	0.063

IF pIOL: iris-fixated phakic intraocular lens; UDVA: uncorrected distance visual acuity; CDVA: corrected distance visual acuity.

**Table 4:** Efficacy and safety indices.

Time (years after the implantation)	1	2	5	12
Efficacy indices (EI)	1.06	1.03	1.03	0.96
Mean postoperative UDVA/mean preoperative CDVA	—	—	—	—
Safety indices (SI)	1.10	1.10	1.10	1.10
Mean postoperative CDVA/mean preoperative CDVA	—	—	—	—

UCVA: uncorrected distance visual acuity; CDVA: corrected distance visual acuity.

**Table 5:** Intraocular pressure.

Time after the IF pIOL implantation	No. of eyes	IOP (mmHg)
0 months	85	14.8 ± 3.0 [8.5; 22.5]
1 months	82	14.5 ± 2.4 [11.5; 22.0]
2 years	61	14.8 ± 2.8 [10.0; 22.5]
5 years	60	15.2 ± 3.0 [9.0; 21.5]

IF pIOL: iris-fixated phakic intraocular lens; No.: number; IOP: intraocular pressure.

**Table 6:** Endothelial cell density after the IF pIOL Implantation.

Time after the IF pIOL implantation	Number of eyes	ECD (cells/mm^2^)	EC loss (%)	Corrected EC loss (%)^*∗*^
1 year	75	2430 ± 312 [1421; 3221]	6.6	6.0
2 years	61	2369 ± 262 [1715; 3009]	9.3	8.1
5 years	60	2175 ± 298 [1286; 2937]	15.8	12.8
12 years	42	2091 ± 312 [1196; 2674]	19.1	11.9

IF pIOL: iris-fixated phakic intraocular lens; No. number; ECD: endothelial cells density; EC loss: endothelial cells loss; ^*∗*^endothelial cells loss corrected to physiological endothelial cells loss.

## Data Availability

The data used to support the conclusions of this study are available from the corresponding author upon request.
